# Dialytic sodium removal in children with acute kidney injury treated with peritoneal dialysis

**DOI:** 10.1007/s00467-025-06861-8

**Published:** 2025-07-08

**Authors:** Peter Nourse, Mignon McCulloch, Ashton Coetzee, Tim Bunchman, Stefano Picca, Dieter Van der Westhuizen, Andre Brooks, Hilton Heydenrych, Brenda Morrow

**Affiliations:** 1 Division of Pediatric Nephrology, Department of Paediatrics and Child Health, Red Cross War Memorial Children’s Hospital, University of Cape Town, Cape Town, South Africa; 2 Children’s Hospital of Richmond, Richmond, VA USA; 3 Educational ambassador for the International Society of Nephrology, Rome, Italy; 4 Division of Chemical Pathology, Department of Pathology, University of Cape Town, Cape Town, South Africa; 5 Division of Cardio-Thoracic Surgery, Department of Surgery, Red Cross War Memorial Children’s Hospital, University of Cape Town, Cape Town, South Africa; 6 Department of Chemical Engineering, University of Cape Town, Cape Town, South Africa; 7 Division of Paediatric Critical Care, Department of Paediatrics and Child Health, University of Cape Town, Cape Town, South Africa

**Keywords:** Peritoneal dialysis, Acute kidney injury, Children, Dialysate salt removal

## Abstract

**Background:**

Dialytic sodium removal (DSR) is an important parameter of peritoneal dialysis (PD) adequacy. The aim of this study was to report the DSR of children with acute kidney injury (AKI) on a standard acute PD prescription and to compare it to that of children on continuous flow peritoneal dialysis (CFPD).

**Methods:**

A secondary analysis of prospectively collected data was performed from a published randomized controlled crossover trial comparing children on conventional PD and CFPD. The conventional PD prescription used: fill volume 20 mL/kg, glucose 2.5%, dwell time 45–60 min. In this study, we described and compared DSR in 15 children with AKI receiving PD and CFPD. Relative ultrafiltration through small pore (UFSP) was also described and compared.

**Results:**

The median (range) weight and age of patients were 5.8 (2.3–14.0) kg and 6 (0.2–14) months. Approximately 8 h of dialysis was received per patient per modality. Results were then extrapolated and expressed per day. The mean ± SD DSR on conventional PD and CFPD were 2.7 ± 6 and 8.4 ± 10 mmol /kg/day, respectively (*P* = 0.02). The mean ± SD sodium dialysate to plasma (D/P) ratio on conventional PD and CFPD were 0.94 ± 0.03 and 0.94 ± 0.04 mmol/mmol (*P* = 1.0). Mean ± SD UFSP to total UF ratios on conventional PD and CFPD were 0.82 ± 0.39 and 0.66 ± 0.51 mL/mL (*P* = 0.14).

**Conclusions:**

This study adds to the limited data on DSR in children on PD for AKI. CFPD removes more salt compared to conventional PD because of increased ultrafiltration (UF). A high percentage of UF was through small pores in both modalities.

**Graphical abstract:**

**Figure:**
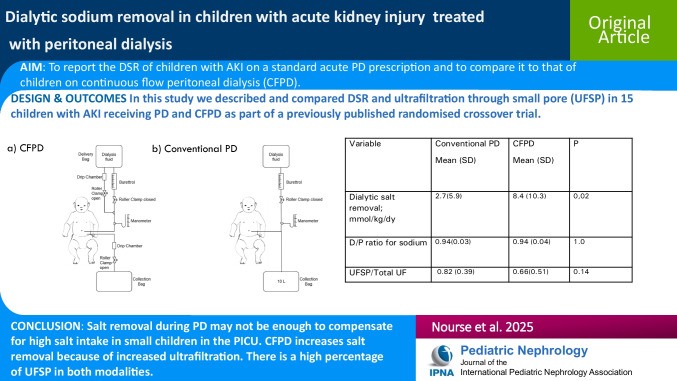
A higher resolution version of the Graphical abstract is available as Supplementary information

**Supplementary Information:**

The online version contains supplementary material available at 10.1007/s00467-025-06861-8.

## Introduction

Dialysate sodium removal (DSR) is an important parameter of dialysis adequacy [1, 2]. Children being treated in pediatric intensive care units (PICU) are particularly vulnerable to excessive fluid and salt delivery. Sodium and fluid are delivered while keeping various monitoring lines open, delivering vasopressors, during the supply of nutrition and correction of acidosis by NaHCO3 and various colloid solutions. In a recent study of critically ill children (not on kidney replacement therapy) in PICU, daily sodium intake was on average 14 mmol/kg/day [3]. This is a lot more than what has been reported to be delivered in critically ill adults (approximately 3 mmol/kg/day) [4]. The extracellular fluid volume is determined by the absolute amounts of sodium and water that are present in the extracellular fluid. Volume is offloaded by natriuresis, a mechanism impaired in critically ill patients, especially when there is any degree of kidney impairment. Thus, these large volumes of saline fluids are more likely to accumulate in the extracellular space. Furthermore, in those children with acute kidney injury (AKI) whose kidneys are still partially working, sodium intake in excess of water (hypertonic) results in increased serum osmolality, which in turn causes antidiuretic hormone release and further free water retention, potentially worsening fluid overload [5–8]. Fluid overload is a common condition in critically ill children with AKI and is known to be independently associated with poor outcomes [9]. Children in PICU on peritoneal dialysis (PD) whose kidneys are not working at all are unable to regulate free water losses or gains via their kidneys and can obviously not drink to thirst, so are completely dependent on the relative input (fluids given) and outputs (dialysis) of salt and water. Imbalances may cause hyper- or hyponatremia. Although many series describing PD use in children with AKI report on initial electrolyte abnormalities, there are no reports that we could find recoding changes in serum sodium concentrations while on PD. Dialysate salt removal and relative free water removal are thus important in terms of extracellular volume overload as well as dysnatremias. In children on peritoneal dialysis for AKI, it is not clear how much salt is removed by the ultrafiltrate, as this has not been adequately described. Goes et al. (2013) reported a median DSR of 33 mmol/day in 31 adult patients on high-volume continuous automated peritoneal dialysis (APD) being treated for AKI [10]. Recent data from the Royal Free Hospital in London reported an average daily sodium removal of 51 mmol/day in 718 adult patients on PD with chronic kidney disease (CKD) [11]. In the only pediatric study to date, Vande Walle et al. reported DSR of 11 mmol/kg/day in children on a PD prescription for AKI [12].

According to the three-pore model [13], ultrafiltration (UF) in PD is due to osmotic pull from the glucose in the peritoneal fluid. This occurs primarily via two pathways: (1) aquaporin transport, which results in free water; and (2) small pore transport, in which electrolytes in the plasma are brought across the membrane via convection in an isotonic manner. Because currently available PD fluids contain 132–134 mmol/L of sodium, the diffusion gradient for sodium is very low thus making the amount of salt transferred by diffusion negligible. From the above, it is clear that the amount of salt removed during a dwell is dependent on the amount of salt transferred by convection through the small pores. The shorter the dwell time and the higher the glucose concentration, the higher the free water fraction of the ultrafiltrate [2] and hence the lower the amount of sodium removed relative to the total UF. This has been demonstrated in adult CKD patients where DSR is lower on automated PD than when on continuous ambulatory PD (CAPD) [11, 14]. The cycling occurring on automated PD is like the type of cycling that occurs in acute PD in children. Furthermore, the currently recommended ISPD acute PD prescription [15] has a shorter dwell time and a lower fill volume than a child on chronic PD. The amount of DSR could therefore be quite different.

The aim of this study was to measure DSR in children with AKI treated with a standard acute PD prescription. Furthermore, the DSR of children on conventional PD was compared to those on continuous flow peritoneal dialysis (CFPD). Secondary aims were to measure the fraction of fluid removed by small pores and compare these between the two dialysis techniques.

## Methods

### Study design

Although this research represents a secondary analysis of data derived from a previously published investigation, the sodium values in the original study were measured with the intention to do the analysis described in this paper. In brief, the original study was a randomized crossover clinical trial involving consecutive eligible patients admitted to a PICU at Red Cross War Memorial Children’s Hospital in Cape Town. Participants were randomly assigned in a 1:1 ratio to one of two treatment sequences: CFPD followed by conventional PD or conventional PD followed by CFPD. The primary aim of the original study was to assess the feasibility of a novel, cost-effective gravity-assisted technique for delivering CFPD and to compare its effectiveness against conventional PD in terms of ultrafiltration and clearances. Comprehensive details regarding the trial design have been previously documented [16].

### Participants, location, and setting

The criteria for inclusion and exclusion have been detailed in prior work [16]. To summarize, eligible participants included PICU patients weighing less than 15 kg who met the Kidney Disease Improving Global Outcomes (KDIGO) criteria for AKI [17, 18] and were deemed by clinicians to require acute dialysis. Patients were excluded if they had contraindications to PD catheter placement, and any patient developing a pleural effusion thought to be secondary to PD was withdrawn from the study. Ethical approval for this research was granted by the University of Cape Town’s Human Research Ethics Committee (HREC 363/2017), and the study protocol was registered with The Pan African Clinical Trials Registry (registration number: PACTR201801003005742). Informed written consent was obtained from the parent or legal guardian of each child.

## Materials and methods

Baseline data collected included age, sex, height, weight, primary admission diagnosis, comorbidities, primary diagnosis, dialysis indication, and AKI classification score.

### Interventions

#### Gravity-assisted CFPD (intervention)

A comprehensive explanation of the gravity-assisted CFPD technique has been previously published [16]. In summary, two 8.5-Fr Multipurpose Fuhrman Cook Catheters (MPCC; Cook Medical Inc., Bloomington, IN, USA) were inserted at the bedside using the Seldinger technique—one serving as an inflow catheter and the other as an outflow catheter. Patients received an initial fill volume of 20 mL/kg of 2.5% lactate PD solution. Once the abdominal cavity was filled, continuous flow was initiated. The inflow rate was set at 50 mL/min/1.73 m^2^, with the outflow rate exceeding the inflow rate by 2.5 mL/min/1.73 m^2^ to account for ultrafiltration (UF). UF volume measurement: After 2 h, and subsequently every 4 h, the inflow was paused while outflow continued for 20 min to ensure complete abdominal drainage. The UF rate was determined using the formula: True UF = (Drainage bag mass at session end—drainage bag mass at session start)—(Delivery bag mass at session initiation—Delivery bag mass at session termination). All spent dialysis fluid was collected, pooled, and sent for laboratory analysis.

### Conventional PD (control)

Conventional PD was carried out following established international protocols [15]. The initial fill volume was 20 mL/kg with a glucose concentration of 2.5%, a dwell time of 45–60 min, an inflow period of 1–10 min, and an outflow duration of 20 min, with adjustments made based on patient needs. Manual dialysis was performed in all patients using a Pediatric PD System (© Fresenius Medical Care South Africa (PTY) Ltd). In conventional PD, UF was quantified by measuring the difference between the volume of fluid introduced and the volume recovered. All spent dialysis fluid was collected, pooled, and submitted for laboratory analysis.

### Laboratory testing

A baseline blood sample was taken and thereafter every 6 h blood was drawn. Spent dialysate was pooled after each dialysis modality for each patient and sent to the laboratory for analysis. Glucose was measured using the enzymatic hexokinase method (CV 1.5%), sodium with an indirect ion-selective electrode method (CV 1.2%), and fluid albumin with an immunoturbidimetric method (CV 2.1%). The assays were carried out in a certified clinical laboratory using validated methodologies for analyzing both blood and fluid specimens. The tests were conducted with the AU480 analyzer (Beckman Coulter Inc., USA) to ensure accuracy and reliability. Quality control parameters were acceptable throughout the study. To address concerns about high glucose concentration in dialysis fluid affecting sodium measurement with an indirect ion-selective electrode [19], we conducted a simple experiment: Dianeal® PD solutions containing 1.5%, 2.5%, and 4.25% glucose monohydrate (corresponding to 75.7, 126.2, and 214.5 mmol/L glucose) were analyzed for sodium on our routine AU480 (Beckman Coulter Inc., USA) auto-analyzer. The approximate sodium concentrations in these solutions as stated by the manufacturer were 141, 132, and 132 mmol/L for Dianeal® 1.5, Dianeal® 2.5, and Dianeal® 4.25, respectively, as indicated by the manufacturer (Baxter International Inc.). Results for the neat PD solutions were 138, 129, and 131 mmol/L, indicating negative differences of 2.1%, 2.3%, and 0.8%, respectively. This difference is smaller than the reference change value for sodium, which is universally estimated at roughly 3% [19, 20] and may at least partly be explained by the coefficient of variation on our analyzer of between 0.9% and 1.5%.

### Outcomes

The following outcomes were analyzed in this study:

1. Dialytic sodium removal
2. Dialysate to plasma (D/P) ratio for sodium
3. Ultrafiltration through small pore (UFSP) to total UF ratio

### Definition of outcomes and calculations

Dialytic sodium removal (mmol) is calculated as: [Volume Dialysate Out (L) × [Na] Dialysate Out (mmol/L)] − [Volume dialysis fluid IN(L) × [Na] Dialysis fluid IN(mmol/L)] [21]. [Na] IN was the concentration according to manufacturer’s specifications and OUT concentration was measured as above. DSR was then expressed in mmol/kg/day.

UFSP was calculated as follows: UFSP (mL) = [dialytic sodium removal (DSR) (mmol) × 1000]/[Na] plasma in mmol/L. UFSP was then expressed in mL/kg/h and expressed as a ratio of total UF also in mL/kg/day. Plasma sodium was the mean plasma sodium while the patient was on the respective dialysis techniques.

UFSP to total UF ratio was calculated by dividing UFSP/total UF. Dialysate to plasma (D/P) sodium ratio was calculated by dividing the dialysate sodium concentration by the mean plasma sodium concentration during the time the patient was on dialysis.

### Statistical analysis

The calculation of sample size was originally based on primary outcomes related to the clearance of creatinine and urea as well as UF, as reported in an earlier paper [16], and is therefore not directly applicable to the current study’s outcomes. Data entry was performed manually using Microsoft Excel (Microsoft, WA, USA) and later transferred to IBM SPSS version 28.01.0 (IBM Corporation, NY, USA) for statistical processing. The normality of continuous variables was assessed using the Shapiro–Wilk test, and descriptive statistics—including measures of central tendency and proportions—were applied based on the distribution and type of variables. For outcome measures, the sodium dialysate-to-plasma ratio, and UFSP, normal distribution was observed, allowing for analysis through parametric methods. In cases where negative ultrafiltration or UFSP occurred (noted only in conventional UF), the UFSP-to-total UF comparison was omitted for those individuals, employing case-wise deletion of missing values. As no period or carry-over effects were identified, paired *t*-tests were used to compare continuous outcome data between the two dialysis methods, enabling an assessment of differences in interventions on a per-participant basis.

## Results

Between 2018 and 2021, consent was obtained from 16 eligible patients. However, one patient was excluded before randomization due to the presence of pleural effusion detected during out-of-study PD, leaving 15 participants for randomization. Of these, nine were assigned to receive CFPD first, while six underwent conventional PD initially. Recruitment was concluded once the required number of participants was reached, as determined by the power calculation for the trial’s primary outcomes. All randomized patients were included in the final intention-to-treat analysis. Baseline patient characteristics are detailed in Table 1. The mean ± SD treatment duration (in hours) was 8.1 ± 5.1 for conventional PD and 8.7 ± 6.6 for CFPD (*P* = 0.71).

**Table 1:** Baseline characteristics of patients

Patient	Age (months)	Wt (kg)	Underlying diagnosis	Serum sodium before dialysis (mmol/L)	Percentage fluid overload (FO) since admission PICU	AKI stage before dialysis
1	0.2	2.3	Diaphragmatic hernia	135	4%	Urine stage 2; Creat stage 2
2	0.33	2.5	TGA post-cardiac surgery	141	Clinically FO ++ 3% from previous day, + + fluid in OT	Urine stage 3; Creat stage 2
3	15	9	Shocked gastroenteritis paracetamol poisoning, liver failure	145	9%	Urine stage 3; Creat stage 2
4	7	5.8	AVSD repair; sepsis; arrhythmia	134	26%	Urine stage 2; Creat stage 3
5	11	8.6	Gastroenteritis, sepsis	128	15%	Urine: Stage 3; Creat stage 3
6	5.4	6.5	Shocked gastroenteritis sepsis, gangrenous foot	161	8%	Urine stage 3; Creat stage 3
7	3	3	TOF post central shunt,	134	9%	Urine stage 0; Creat stage 3
8	0.26	3.3	TGA post-surgery	135	22%	Urine stage 3; Creat stage 2
9	11.5	6.71	TOF post-surgery repair	136	9%	Urine stage 0; Creat stage 2
10	0.5	2.7	TGA post-surgery repair	138	24%	urine stage 1; Creat stage 0
11	10	10	Septic shock	134	Clinically FO ++ (transferred from another hospital for dialysis)	Urine stage 2; Creat stage 3
12	1.5	3.5	Hemi truncus arteriosus; congenital syphilis	123	6% FO	Creat stage 3 urine stage 0
13	35	14	HUS diarrhea associated	128	Clinically FO ++ (transferred from another hospital for dialysis)	Urine stage 2; Creat stage 3
14	6	5	TAPVD post-septostomy	134	5%	Urine stage 0; Creat stage 3
15	14	12	Myocarditis	155	2%	Urine stage 2; Creat stage 2
Median(range)	6 (0.2–14)	5.8 (2.3–14)				

*AVSD*, atrio-ventricular septal defect; *HUS*, hemolytic uremic syndrome; *PICU*, pediatric intensive care unit; *TAPVD*, total anomalous pulmonary venous drainage; *TGA*, transposition of the great arteries; *TOF*, tetralogy of Fallot

### Outcome and estimations

The initial analysis demonstrated that the sequence of assignment did not influence changes in ultrafiltration. This finding is relevant to this study, as DSR is dependent on ultrafiltration. Additionally, to control for patient-related variables over time, baseline characteristics were compared across each modality for the entire duration that each patient remained on that modality. The variables assessed included ventilatory settings, heart rate, blood pressure, and blood oxygen saturation. No significant differences were observed. Both analyses are detailed in our previous publication on this patient cohort [16]. Four patients were excluded from the analysis comparing UFSP/total UF because of negative UF on conventional PD. Table 2 presents the outcomes and comparison of DSR, UFSP/total UF ratio, and D/P sodium ratio for conventional PD and CFPD. DSR on CFPD was significantly higher than on conventional PD. The fractions of UFSP/total UF were 82% for conventional PD and 66% for CFPD, but the difference was not statistically significantly different. D/P sodium ratios were also not significantly different between techniques.

**Table 2:** Dialytic salt removal and ultrafiltration through small pores

Variable	Conventional PD Mean (SD)	CFPD Mean (SD)	*P*
Dialytic salt removal, mmol/kg/day	2.7 (5.9)	8.4 (10.3)	0.02
D/P ratio for sodium	0.94 (0.03)	0.94 (0.04)	1.0
UFSP/total UF	0.82 (0.39)	0.66 (0.51)	0.14
UFSP (mL/kg/h)	1.6	3.25	
Total UF (mL/kg/h)	1.8	5.0	

*CFPD*, continuous flow peritoneal dialysis; *D/P*, dialysate over plasma; *PD*, peritoneal dialysis; *UF*, ultrafiltration; *UFSP*, ultrafiltration through small pores

## Discussion

This paper adds to the very sparse data on dialytic sodium removal in children treated with PD for AKI. The DSR in conventional PD of 2.7 mmol/kg/day was less than the 11 mmol/kg/day described in eight children by Vande Walle in 2005 [12]. Sodium removal in critically ill children on PD is important as demonstrated by the large sodium load delivered in the Langer study (2020) of 14 mmol/kg/day [3]. This is very different than children with congenital kidney abnormalities on maintenance PD for CKD who are often polyuric and salt losers [22]. The aim of the PD prescription in terms of salt removal is therefore very different in maintenance PD for children with CKD compared to critically ill children with AKI. In adult patients on high-volume PD for AKI, Goes et al. (2013) described a median salt removal in 31 adult patients with AKI treated with high volume PD of 33 mmol/day. When corrected for an average adult body weight of 67 kg, to compare to pediatric data, this is equivalent to only 0.5 mmol/kg/day. Adult ICU patients, however, are not as salt loaded as pediatric patients [4] and so low salt removal may be less of a problem in this population. In a small pediatric study in children with AKI, low sodium concentration PD fluids were shown to enhance sodium removal [12]. Low-sodium solutions have also been shown to increase sodium removal in adult patients with CKD [23], and this possible solution to salt removal should be explored in further pediatric studies. Using bicarbonate-based PD fluids in small babies who are unable to metabolize lactate may better correct acidosis, thus limiting the amount of sodium given with sodium bicarbonate infusions. Sodium removal on CFPD was significantly higher than on conventional PD, and this may also potentially be a solution to this problem; however, further studies are needed to confirm these findings. The relative increase in ultrafiltration (and hence DSR) was like the results of our previous studies [24, 25] when we used pumps from CVVH machines to drive the fluid through the abdomen. In the current study, however, we used half the flow rate indicating the faster rates may be unnecessary. Ultrafiltration achieved was not as high as reported in adult studies using CFPD in patients with CKD [26–28]. Lower fill volumes and flow rates as well as the acute illness of the children which may affect perfusion of the peritoneal membrane could account for this.

The ratio of UFSP to total UF in children on conventional PD with a 1-h dwell was 82% of total UF, which was also reflected by the D/P sodium ratio of 94%. This is like the 85.5% of total UF described by Cano (2013) in a 1-h mini-PET test in 16 children on maintenance PD for CKD [29]. In a very recent study by Tokunaga et al. (2024) [30], high sodium sieving was described especially in the first hour in children on chronic PD. The authors of this study demonstrated a 1-h D/P ratio for sodium from 0.56 to 0.62 depending on transporter status. In adult patients on maintenance PD for CKD, La Milia (2006) [31] showed UFSP to be 55–65% of total UF in the early part of a 4-h dwell. Because of the short dwell times used in small children with AKI, we expected the fraction of free water to be higher than what was demonstrated [2, 13]. It appears however that most of the UF in our patients was via small pores with less sodium sieving taking place. An explanation for this may be that many of these small patients are high transporters, firstly because of low fill volumes prescribed in acute PD and also possibly due to inflamed peritoneal membranes. We have tried to demonstrate this in a previous study [32]. The dwell time therefore may not be long enough to demonstrate the effect of sodium sieving. Once sodium sieving has occurred and the glucose gradient has dissipated, the dialysis fluid sodium concentration is decreased so that there is diffusion of sodium down the concentration gradient from the blood to the dialysis fluid before the fill volume is drained. This effect has been demonstrated in adult patients where automated peritoneal dialysis has increased sodium sieving compared with CAPD [11, 14]. The sodium concentration of the drained fluid is thus higher at the end of the dwell time than earlier on, and this effect masks sodium sieving. The sodium sieving while the patients were on CFPD was more than on conventional PD. This is to be expected as the continuous flow has the same effect as very short dwells, where the free water ultrafiltrate is carried off before there is time for equilibration. Although there was no significant difference in the UFSP to total ultrafiltration ratio between the two dialysis modalities, the study was not powered for this outcome. The DSR was increased with CFPD despite the increased sodium sieving, and this is probably only because of the increased UF on this modality [16]. Low sodium fluids may be especially beneficial in patients on CFPD to enhance sodium removal. Both our study in which all but one patient was an infant, and in the Cano study where the average age of the patients was 3.5 years [29] was the free water fraction lower than in adult studies. We know the peritoneal membrane changes quite significantly in terms of blood vessel and lymphatic density from infancy to childhood and then into adulthood [33]. One could postulate that there are also differences in aquaporin density in children compared to adult patients, but this has not been proven.

This study had limitations due to its single-center design and small heterogeneous sample size, primarily consisting of infants with various underlying conditions and degrees of illness severity. The study might have lacked sufficient power for secondary comparisons between CFPD and PD, which should be considered when interpreting the results. Additionally, the approximately 8-h duration for each dialysis method might not accurately reflect the true dialysis salt removal over the entire treatment period. It is important to acknowledge that sodium measurement in dialysate is subject to several possible methodological pitfalls, including differences between direct and indirect ion-selective electrode (ISE) techniques, matrix effects due to varying ionic strength, and potential dilution errors which are inherent to the indirect sodium ISE measurement method. These can lead to discrepancies in measured versus actual sodium concentrations. As highlighted by Lefevre (2023) [19], calibration methods and instrument-specific biases must be considered when interpreting dialysate sodium levels. We attempted to address these concerns by the experiments that we have described in the methods. We acknowledge that this is not sufficient in itself to conclude that the method is reliable at the individual level. We acknowledge this as a limitation but also highlight it as an area for further research. Rather than only measuring DSR as an outcome, it would also be better to measure fluid status and dysnatremias as outcomes. This would require larger numbers and would need to be powered for this.

## Conclusion

This study adds to the limited data on DSR in children receiving PD for AKI. CFPD increases DSR in children and may be a useful modality in patients receiving a high salt load in PICU. Most UF in this pediatric cohort was through small pores, with only a small amount of free water transport.

## Supplementary Information

Below is the link to the electronic supplementary material.

Graphical abstract (PPTX 132 KB)

## Data Availability

Individual participant data that underlie the results reported in this article will be made available after deidentification. Availability will follow publication, no end date. The data will be made available to researchers whose use of the data has been approved by an independent review committee. The purpose of the data access will be to achieve the aims in the research proposal. Proposals should be directed to peter.nourse@uct.ac.za. To gain access, data requesters will need to sign a data transfer agreement with the University of Cape Town.
