# Supplementary material for: Dialytic sodium removal in children with acute kidney injury treated with peritoneal dialysis
Source: Pediatr Nephrol. 2025 Jul 8;41(2):557–64. doi: 10.1007/s00467-025-06861-8 (PMC12727783; doi:10.1007/s00467-025-06861-8)
Supplement: Supplementary file 1 — Graphical abstract (PPTX 132 KB) [file 467_2025_6861_MOESM1_ESM.pptx]

## Slide 1
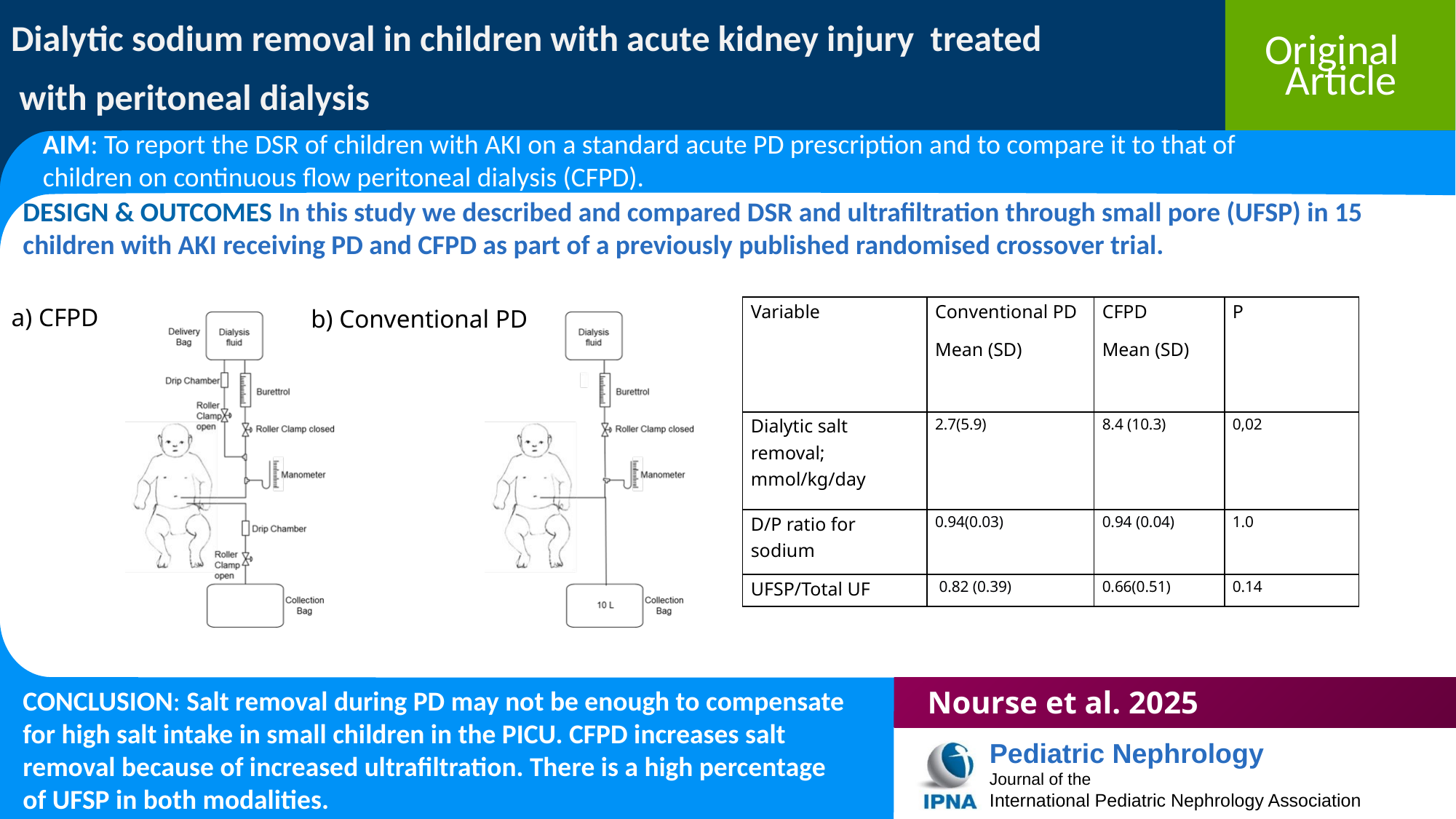

Dialytic sodium removal in children with acute kidney injury treated
 with peritoneal dialysis
AIM: To report the DSR of children with AKI on a standard acute PD prescription and to compare it to that of children on continuous flow peritoneal dialysis (CFPD).
DESIGN & OUTCOMES In this study we described and compared DSR and ultrafiltration through small pore (UFSP) in 15 children with AKI receiving PD and CFPD as part of a previously published randomised crossover trial.
a) CFPD
| Variable | Conventional PD Mean (SD) | CFPD Mean (SD) | P |
| --- | --- | --- | --- |
| Dialytic salt removal; mmol/kg/day | 2.7(5.9) | 8.4 (10.3) | 0,02 |
| D/P ratio for sodium | 0.94(0.03) | 0.94 (0.04) | 1.0 |
| UFSP/Total UF | 0.82 (0.39) | 0.66(0.51) | 0.14 |
b) Conventional PD
CONCLUSION: Salt removal during PD may not be enough to compensate for high salt intake in small children in the PICU. CFPD increases salt removal because of increased ultrafiltration. There is a high percentage of UFSP in both modalities.
Nourse et al. 2025
